# Single-Unit Activity in the Medial Prefrontal Cortex during Immediate and Delayed Extinction of Fear in Rats

**DOI:** 10.1371/journal.pone.0011971

**Published:** 2010-08-05

**Authors:** Chun-hui Chang, Joshua D. Berke, Stephen Maren

**Affiliations:** 1 Department of Psychology, University of Michigan, Ann Arbor, Michigan, United States of America; 2 Neuroscience Program, University of Michigan, Ann Arbor, Michigan, United States of America; University of New South Wales, Australia

## Abstract

Delivering extinction trials minutes after fear conditioning yields only a short-term fear suppression that fully recovers the following day. Because extinction has been reported to increase CS-evoked spike firing and spontaneous bursting in the infralimbic (IL) division of the medial prefrontal cortex (mPFC), we explored the possibility that this immediate extinction deficit is related to altered mPFC function. Single-units were simultaneously recorded in rats from neurons in IL and the prelimbic (PrL) division of the mPFC during an extinction session conducted 10 minutes (immediate) or 24 hours (delayed) after auditory fear conditioning. In contrast to previous reports, IL neurons exhibited CS-evoked responses early in extinction training in both immediate and delayed conditions and these responses decreased in magnitude over the course of extinction training. During the retention test, CS-evoked firing in IL was significantly greater in animals that failed to acquire extinction. Spontaneous bursting during the extinction and test sessions was also different in the immediate and delayed groups. There were no group differences in PrL activity during extinction or retention testing. Alterations in both spontaneous and CS-evoked neuronal activity in the IL may contribute to the immediate extinction deficit.

## Introduction

Failure to extinguish fear memory is a core feature of several anxiety disorders [1], [2], [3], [4], [5]. In rats, extinction has been studied extensively using Pavlovian fear conditioning procedures [6], [7], [8]. After a conditioned stimulus (CS) has been paired with an aversive unconditioned stimulus (US), repeated presentation of the CS alone leads to a loss of conditioned fear response. This loss of fear is fragile, recovering with the passage of time and with changes in context [9], [10], [11]. Hence, extinction procedures do not erase fear memory, but yield a new safety memory that inhibits fear under certain conditions. In recent years, considerable progress has been made in understanding the neural circuitry underlying fear extinction, which involves a distributed neural circuit including the amygdala, medial prefrontal cortex (mPFC), and hippocampus [6], [8], [12]. Yet although considerable progress has been made in understanding the behavioral and neurobiological mechanisms underlying extinction in experimental models [9], [11], [13], less progress has been made in understanding the nature and causes of extinction impairments that contribute to psychopathology in humans. Interestingly, we have found that a recently acquired fear memory is especially difficult to extinguish [14], [15], which may provide a model to explore the resistance to extinction observed in patients with post-traumatic stress disorder [16].

Recently, two regions of the mPFC have been implicated in the expression of fear. The infralimbic division (IL) projects to inhibitory neurons in the amygdala involved in suppressing fear after extinction [8], [17], [18], whereas the prelimbic division (PrL) projects to projection neurons in the amygdala involved in fear expression [19], [20], [21]. Several lines of data implicate the IL in the acquisition and extinction of fear memories. Although studies employing IL lesions have not yielded consistent effects on fear extinction [22], [23], pharmacological manipulation [24] or electrical stimulation [25], [26] of IL influences extinction memory. Moreover, electrophysiological correlates of extinction develop in the IL, including increases in CS-evoked single-unit responses [26] and increases in spontaneous bursting [27] after extinction training.

In the light of this circuitry, we hypothesize that neuronal activity in the IL that is involved in the acquisition of long-term extinction memory may be disrupted under immediate extinction conditions [24]. Consistent with this possibility, it has recently been reported that immediate extinction yields fewer Fos immunoreactive neurons in IL, and electrical stimulation of IL during immediate extinction rescues the deficit [28]. To further explore this hypothesis, we characterized neuronal activity in both the IL and PrL during immediate and delayed extinction using high-density electrophysiological recordings in awake, behaving rats. We found that successful extinction was associated with attenuation of tone-responsivity and the development of neuronal bursting in the IL; this effect was attenuated in rats undergoing immediate extinction. These data suggest that IL dysfunction accounts for the immediate extinction deficit, and reveal a therapeutic target for overcoming extinction deficits in humans with PTSD.

## Results

### Histology

**Figure 1:**
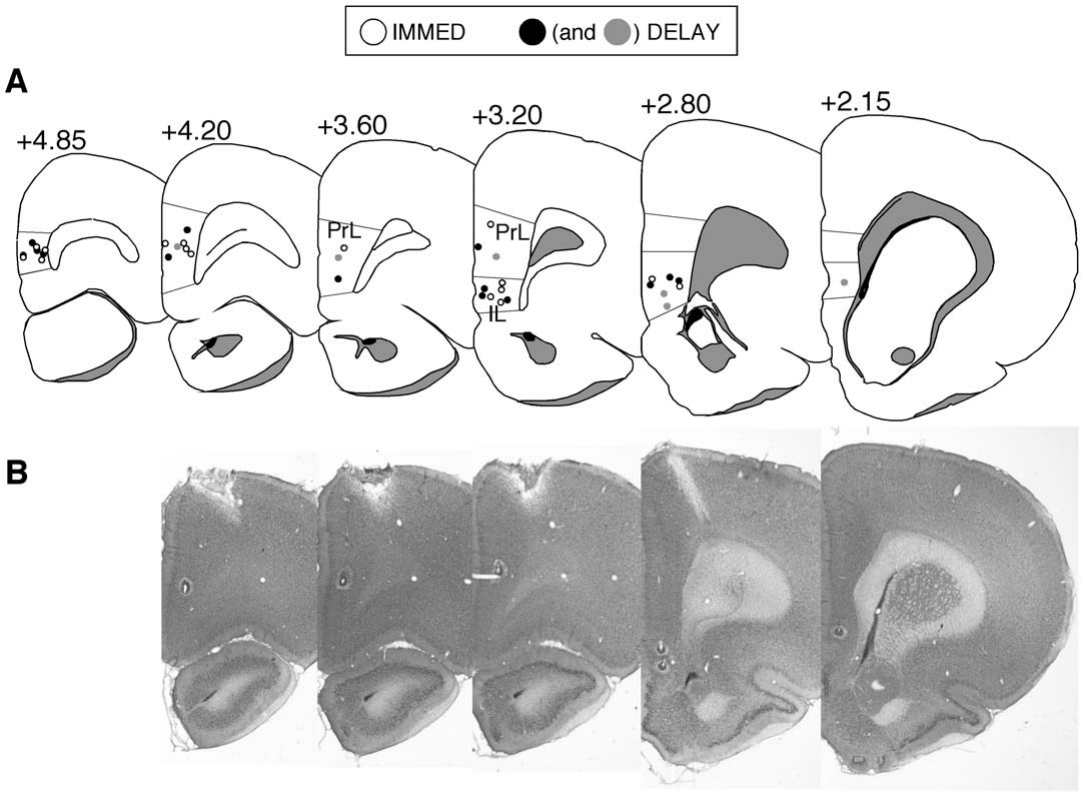
Anatomical placement of tetrodes. (A) Coronal sections representing all of the tetrode placements included in the data analysis. (B) Serial sections from one DELAY animal showing tetrodes in both the IL and PrL; these placements are shown as filled gray circles in (A).

Unit recording sites in IL and PrL are illustrated in Figure 1. The total number of neurons recorded in each area and behavioral session is summarized in Table 1. All units were treated as independent neurons across each of the behavioral sessions.

**Table 1:** Number of neurons recorded in each behavior session.

		Behavior session		
Group	Brain structure	BL	EXT	TEST
DELAY	IL	28	43	47
	PrL	52	57	70
IMMED	IL	36	44	47
	PrL	43	50	55

### Behavior

Rats were submitted to four phases of training: baseline (BL), fear conditioning, extinction (EXT), and a retention test (TEST). For the baseline session, all rats were presented with ten tone conditional stimuli (CS; 80dB, 2 sec, 10kHz, inter-trial interval = 1 min) in a novel context. They next received fear conditioning with five tone-shock (0.5s, 1mA) pairings delivered in another novel context. Depending on their group assignments, the rats were then extinguished with 50 tones either 15 minutes (IMMED, n = 4) or 24 hours (DELAY, n = 4) in the baseline context. Twenty-four hours after extinction all rats received a retention test consisting of 10 CS-alone presentations in the baseline context. Unit activity was recorded during both the 2-s tone CS period and the inter-trial intervals during BL, EXT, and TEST.

**Figure 2:**
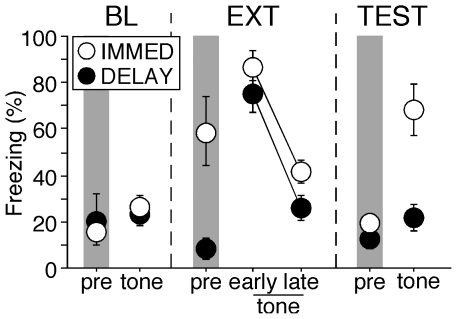
Percentage of freezing (mean ± SEM) during the baseline (BL), extinction (EXT), and test (TEST) sessions in rats undergoing immediate (IMMED) or delayed (DELAY) extinction. Freezing was significantly higher in IMMED rats compared to their DELAY controls prior to extinction (pre-period in EXT session) and during the retention test (p<0.05). Data were averaged across the 10-min pre-CS period or 10 CS trials (first and last 10 trials for early and late EXT periods).

Freezing behavior during each session is shown in Figure 2. As we have previously reported [14], [15], rats receiving immediate extinction exhibited normal short-term fear suppression but recovered this fear the following day. An ANOVA performed on freezing behavior during the tone periods revealed significant main effects of group [F(1,6) = 15.1, p<0.01] and behavioral phase [F(3,18) = 25.9, p<0.0001], and a significant interaction between the two [F(3,18) = 3.8, p<0.05]. *Post hoc* comparisons revealed that freezing behavior between the two groups was equivalently low during the BL session. As we have previously observed [14], [15], IMMED rats exhibited significantly higher levels of freezing during the pre-CS period of the extinction session [F(1,6) = 10.5, p = 0.02]. This pre-CS freezing may be due to sensitization of fear by recent footshock or generalized fear to the extinction context. Nonetheless, presentation of the CS during the extinction session evoked robust freezing behavior relative to the pre-CS period in both groups of rats, and this response decreased equivalently across the session in all rats [ps>0.05]. During the TEST session, freezing among rats in the IMMED condition was significantly higher than that in the DELAY condition [p<0.05], revealing that they exhibited weaker long-term extinction than rats in the delay condition.

### Electrophysiology

#### CS-evoked spike firing

Studies have shown that IL, but not PrL, neurons exhibit increases in CS-evoked firing after extinction training that correlate with fear suppression [12], [26]. Moreover, electrical stimulation of the IL that mimics CS-evoked spike firing suppresses freezing in rats have not been extinguished [25], [26]. We thus hypothesized that impaired extinction in the IMMED rats might be reflected by a failure of IL neurons to increment their firing to an extinguished CS.

As shown in Table 1, we recorded ∼40 units in the IL (mean n = 41, range from 28 to 47) and ∼50 units in the PrL (mean n = 55, range from 43 to 70) across three behavioral sessions. Waveform analysis [29] suggested that all of the neurons we recorded were primarily projections neurons with wide half-peak and peak-valley durations (mean = 164.5±1.0 µs and 453.9±3.2 µs, respectively). Two neurons recorded in the PrL had narrow spike widths (half peak<120 µs, peak-valley<200 µs) and were presumed to be interneurons; they were excluded from further analyses. All neurons displayed low spontaneous firing rates (<3 Hz), which is characteristic of projection neurons [29], [30], [31], [32].

**Figure 3:**
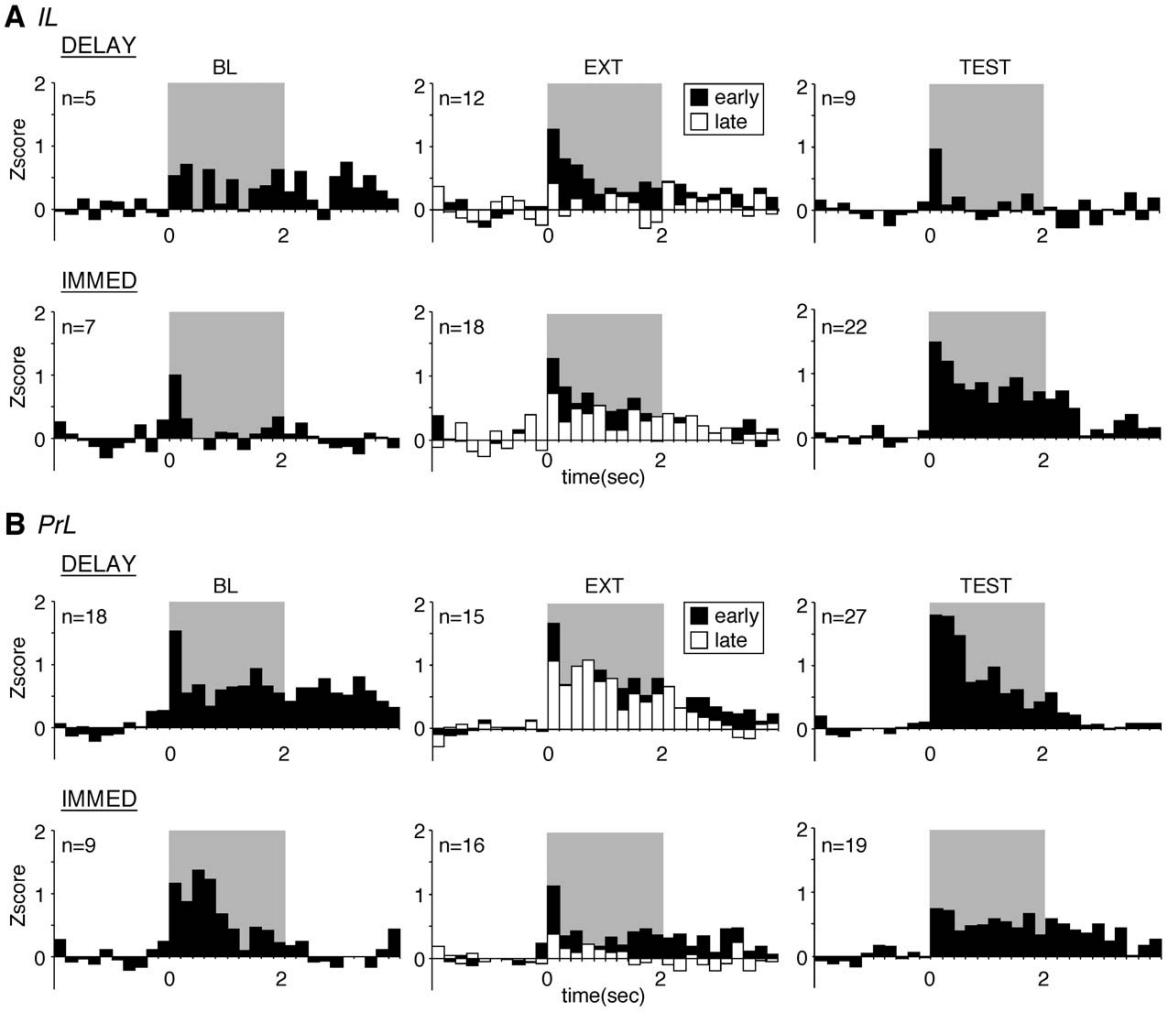
Peri-event time histograms illustrating CS-evoked activity in the IL (A) and PrL (B) during behavioral training. The number of neurons contributing to each average (Z>0 within 200 ms) is indicated in the panels. (A) For the IL, there was no difference between the IMMED and DELAY groups in firing to tones during the baseline (BL) session (p>0.05) and early extinction (EXT). During the extinction session, only DELAY rats decreased their firing to the CS (p<0.05). Firing to tone CSs was significantly higher in IMMED rats compared to DELAY rats during the test session (TEST, p<0.05). (B) For the PrL, there was no difference between groups in any of the behavioral phases. The 2-sec tone CS period is shaded in gray.

We characterized CS-evoked single-unit activity in both IL and PrL neurons during each behavioral session. Overall, the number of CS-responsive neurons in both the IL and PrL was low. Only 13 out of 245 neurons in the IL and 13 out of 327 neurons in the PrL met our standard criterion (z>3 in any post-CS bin within 200 ms of CS onset) for tone responsivity [33], [34], [35]. We therefore softened the criteria for CS-responsiveness and focused our analysis on neurons that showed any increase in firing to the tone CS (*z*>0 within 200 ms of CS onset). Among all the neurons recorded, only about 30% exhibited tone-responsivity in each of the behavioral phase (IL, mean CS-responsive neurons = 29%, range from 18% to 47%; PrL, mean CS-responsive neurons = 31%, range from 21% to 39%). Average peri-event time histograms of spike firing in these neurons are shown in Figure 3 and the analyses of these data focused on average activity across the 2-sec tone CS. Moreover, because different populations of neurons were sampled across each of the three test sessions, our analyses focused on between-group comparisons of activity in each session, rather than within-group comparisons across sessions (although we were able to make within-group comparisons of activity during the extinction session examining early versus late trials in each group).

For IL, there was no significant difference in CS-evoked spike firing among the groups [F(1,9)<1] during the BL session (Figure 3A). During the EXT session, both IMMED and DELAY rats showed substantial CS-evoked firing early in the extinction session, which decreased during the course of the EXT session and paralleled decreases in CS-evoked freezing (Figure 2). There was no significant difference in CS-evoked firing among the groups [F(1,28)<1], but there was a main effect of trials (early>late) [F(1,28) = 6.9, p<0.01]. This main effect was largely carried by the DELAY animals insofar as planned comparisons revealed that CS-evoked firing reliably decreased only in that group [F(1,11) = 6.1, p<0.05]. During the TEST session, CS-evoked firing was significantly lower in DELAY rats relative to IMMED rats [F(1,29) = 4.5, p<0.05]. Thus, animals in the DELAY group that successfully extinguished fear exhibited *lower* levels of CS-evoked firing than animals in the IMMED group that did not exhibit extinction. Indeed, CS-evoked spike firing in the IL appeared to correlate more strongly with the expression of fear than extinction. This outcome was not expected based on previously published work [26].

In the prelimbic division of the mPFC, CS-evoked responses were generally more robust in amplitude than in the IL, and PrL neurons exhibited CS-evoked firing in all of the behavioral sessions (Figure 3B). However, in contrast to the IL, there were no significant differences between the immediate and delayed extinction groups in any of these sessions [BL, F(1,25)<1; EXT, F(1,29) = 1.6, p = 0.22; TEST, F(1,44)<1]. During the extinction session, there was also no difference in the CS-evoked response from early to late trials [F(1, 29)<1] nor was there a significant group×trial interaction [F(1,29)<1]. Together, these results indicate that the activity of neurons in the IL, but not PrL, correlate with the immediate extinction deficit. During the extinction session, IL neurons showed within-extinction decreases in CS-evoked activity that paralleled within-session decreases in freezing and they showed different levels of CS-evoked firing during the retention test that reflected different levels of freezing to the CS during the test.

#### Spike bursting

Previous studies have shown that spike bursting in the IL is a neural correlate of extinction in rats [27], [36]. Quirk and colleagues have defined IL bursting as the occurrence of three or more consecutive spikes with an inter-spike interval (ISI) of less than 30 ms between the first two spikes and less than 50 ms for subsequent spikes [27], [37]. Thus, we examined whether extinction alters the frequency of bursting in the mPFC. We focused the analysis on trial-related bursting events occurring during the BL, EXT, and TEST sessions.

**Figure 4:**
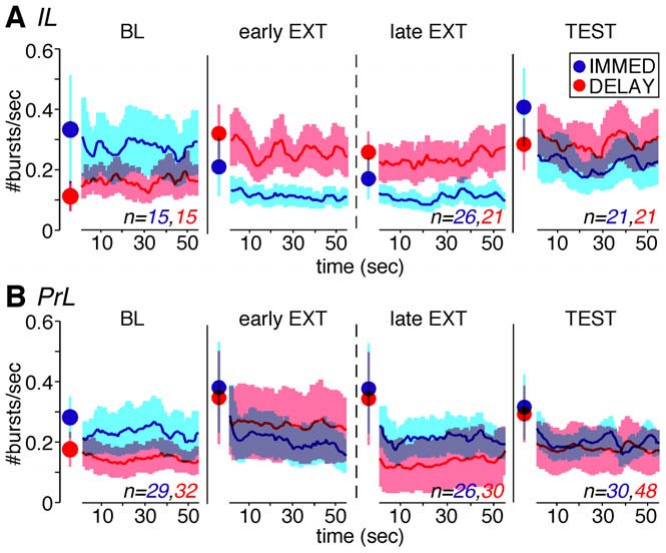
Neuronal bursting in the IL (A) and the PrL (B) divisions of the medial prefrontal cortex. (A) The frequency of IL bursting (mean ± SEM) during the 2-s CS (filled circles) and the 1-min ITI (moving average of 5 sec) is shown for rats in the IMMED and DELAY groups; the number of neurons showing bursting in each session is indicated in the panels. During extinction, bursting was greater in the DELAY rats. (B) There was no significant difference in PrL bursting between the IMMED and DELAY groups for trial-related bursting across different behavioral phases (all ps>0.05).

To elucidate whether IL bursting is linked to CS onset, we quantified trial-related bursting during both the 2-s CS and the 1-min inter-trial interval (ITI) following the CS in each behavioral phase. Consistent with an earlier report [27], bursts in the IL were infrequent (∼0.2 Hz). Among all of the neurons recorded, only about half exhibited bursting during the CS or early in the ITI (within 5s) in each behavioral phase (mean number of bursting neurons = 54%, range from 42% to 69%), and thus we focused our analysis on this sub-population. Trial-related IL bursting was equivalent in the IMMED and DELAY groups during the baseline session [Figure 4A; F(1, 28) = 1.4 and 1.04, tone and ITI respectively; both ps>0.25]. However, differences in bursting between the two groups emerged in the extinction session: DELAY rats exhibited higher levels of bursting than IMMED rats. This was the result of a loss of bursting in the IMMED rats soon after CS offset. That is, there was no significant difference between the groups during the CS period [both F(1,45)s<1 for the early and late trials], but significant differences emerged during the ITI periods [F(1,45) = 5.1, p<0.05 and F(1,45) = 4.2, p<0.05 for the early and late periods, respectively]. During the test session, IMMED rats exhibited a pattern of bursting that was similar to that in the DELAY group in the EXT session. Of course, during the test session, the IMMED rats were receiving an extinction session 24 hours after conditioning, which mirrors the experience of the DELAY rats during the extinction session. There was no significant difference between IMMED and DELAY rats during either the tone or ITI periods during the test session [both F(1, 40)s<1]. Theses data suggest that rats undergoing immediate extinction exhibited less bursting during extinction training, and this was correlated with recovery of fear during retention test. In contrast to the IL, there were no group differences in bursting in the PrL in any behavioral phase [Figure 4B; all ps>0.05]. Moreover, there was no significant difference in spontaneous firing rate between groups in either IL or PrL in any of the behavioral sessions [all ps>0.05]. Hence, the group difference in IL bursting during extinction cannot be attributed to changes in overall firing rate.

## Discussion

In the present study, we used electrophysiological recordings in the medial prefrontal cortex to examine the neural correlates of the immediate extinction deficit in rats. Our results reveal that rats undergoing immediate extinction exhibited minimal suppression of CS-evoked firing in the IL during extinction training, and maintain CS-responsiveness during a retention test twenty-four hours later. Despite elevated CS-evoked responses, IL neurons in rats undergoing immediate extinction exhibited similar levels of bursting to the CS during extinction training, although these burst responses were significantly attenuated during the inter-trial intervals. Altered CS-evoked neuronal firing and intertrial bursting in the IL may contribute to the failure of rats undergoing immediate extinction to maintain fear suppression the following day.

In previous studies, increases in IL bursting have been reported during the consolidation of extinction memory after extinction training was complete [27], [36]. Together with the sensitivity of extinction to post-training pharmacological manipulations of IL [38], these data have been argued to support a role for IL bursting in the consolidation of extinction memories [27], [36], [39]. Our current data reveal that increases in IL bursting also occur during extinction training, but only in those rats that ultimately express a long-term extinction memory. This suggests that the successful acquisition of extinction may require an increase in IL bursting both during extinction training, and in the post-extinction period to foster the consolidation of extinction. As in previous reports [27], the IL bursts we recorded were infrequent (∼0.1 Hz). Although infrequent, these bursts may foster local synaptic plasticity in the IL [27], [40] and facilitate the integration of hippocampal and amygdala inputs [11], [41], [42], [43], [44], [45] in the mPFC. IL bursting might also facilitate the activity of inhibitory intercalated cells in the amygdala that are involved in the inhibition of fear [8], [46].

In addition to the bursting, we found that CS-evoked activity in the IL (at least among those neurons that exhibited an excitatory response to the CS) did not dissipate during the course of immediate extinction training, unlike that in rats undergoing delayed extinction. Furthermore, CS-evoked responses in IL were significantly greater during the retention test in animals that failed to extinguish. This result was unexpected insofar as an earlier report found that CS-evoked responses in the IL were minimal before extinction training, and increased in magnitude after extinction [12], [26]. Indeed, the pattern of CS-evoked firing that we have observed in the present study is more consistent with the firing properties of a subpopulation of tone-responsive neurons recently described in the PrL [21]. The reasons for these disparities are not clear, but it suggests that CS-evoked activity in IL neurons may reflect both the acquisition of conditional fear, as well as its extinction. In either case, the present data suggest that neuronal activity in the IL is altered in rats that fail to extinguish fear relative to those that extinguish normally.

What might account for changes in mPFC activity in rats undergoing immediate extinction? One possibility is that the stress engendered by a recent traumatic event, i.e., fear conditioning, might yield the immediate extinction deficit. Stress-induced arousal appears to compromise the function of mPFC circuits involved in extinction learning. Several studies have found that stress impairs IL function and impairs extinction [3], [47], [48], [49], which may be related to hyperactivity of the amygdala circuits that project to the mPFC [50]. Hence, the neural circuits involved in the generation and suppression of fear may antagonize one another, with the subcortical expression of fear responses dominating the acute response to trauma and the emergence of cortical fear suppression appearing only after the acute stressor has subsided. Another possibility is that animals undergoing immediate extinction do not temporally parse the conditioning and extinction contingencies due to the relative recency of the two events. Thus, IL neurons may maintain their response to the tone as if the conditioning contingency is still in place.

Ultimately, there is considerable debate in the clinical literature about when therapeutic interventions should be attempted after psychological trauma [51], [52], [53], [54], [55], [56]. Recent work in animal models suggests that early interventions do not yield long-term fear suppression [14], [15], [57], [58], [59]. We now show that the function of the neural circuits involved in encoding extinction memory is altered shortly after an acute trauma. This study suggests a potential brain target for combined early interventions with pharmacological treatment after trauma to increase the likelihood of long-term fear suppression.

## Materials and Methods

### Subjects

The subjects were eight male Long-Evans rats (>400 g; Blue Spruce) obtained from a commercial supplier (Harlan Sprague Dawley, USA). They were singly housed in individual cages on a 14-h light/10-h dark cycle (lights on at 7:00 am) and allowed food and water ad libitum. During the first 5 days, they were handled for 10 sec each day to habituate them to the experimenter. All experiments were carried out in accordance with guidelines approved by the University of Michigan University Committee on Use and Care of Animals.

### Electrophysiology

Each rat was implanted with a head assembly containing 18 individually drivable tetrodes [60] aimed at the medial prefrontal cortex (infralimbic and prelimbic cortex). Skull screws were implanted for reference (1 mm posterior to lambda) and ground (posterior lateral skull ridge). Several additional screws were implanted as anchors, and the assembly was affixed to the skull with dental acrylic.

Neuronal and behavioral data acquisition was performed using a 96-channel amplifier system (Boston University Electronics Design Facility) and acquired on-line using SciWorks software (DataWave Technologies, Longmont, CO). Single-unit activity was amplified (10,000×), filtered (300 Hz to 6 kHz), and digitized (31.25 kHz) and written to disk. Tetrodes were progressively lowered into target area across several days and were left in place at least three days prior to the starting of behavioral procedures (roughly ten days after surgery). Tetrode placement was performed on a swivel stool outside of the recording chamber that was to be used for the behavioral experiments. Neuronal and behavioral data were continuously acquired during each behavioral session and stored for further analyses.

Single units with (signal-to-noise ratio>2) were detected off-line using SciWorks, and then manually discriminated and clustered using Offline Sorter (Plexon Inc., Dallas, TX). Data were then imported into NeuroExplorer (NEX Technologies, Littleton, MA) for analyses of firing rate, inter-spike intervals, peri-event time histograms, and bursting.

### Behavioral apparatus

One standard rodent conditioning chamber (30×24×21 cm; MED-Associates; as described in experiment 2) was modified to accommodate electrophysiological recording. It rested on a load-cell platform that was used to record chamber displacement in response to each rat's motor activity. The load cell amplifier output was digitized at 5 Hz and acquired on-line using SciWorks (DataWave Technologies, Longmont, CO).

Two distinct contexts were used in this experiment. For the first context (context A), a 15 W houselight mounted opposite the speaker was turned on, and room lights remained on. The chamber was cleaned with a 1% acetic acid solution. To provide a distinct odor, a stainless steel pan containing a thin layer of this solution was placed underneath the grid floors before the rat was placed inside. The ventilation fan in chest supplied background noise (65 dB). For the second context (context B), the room lights were turned dim and the chamber houselight was turned off. Additionally, the door on the sound-attenuating cabinet was closed, the ventilation fan was turned off, and the chamber was cleaned with 1% ammonium hydroxide solution. Also, stainless steel pans containing a thin layer of the same solution was placed underneath the grid floors before the rat was placed inside to provide a distinct odor.

### Behavioral procedures

Rats were submitted to four phases of training: baseline (BL), fear conditioning, extinction (EXT), and a retention test (TEST). Unit activities were acquired during the BL, EXT, and TEST sessions

On Day 1, rats received 10 tone-alone (2 sec, 80 dB, 10kHz) presentations during the BL session after placement in the recording chamber (context A). Rats then received five tone-footshock (1 mA, 0.5 sec) trials (60 sec inter-trial interval (ITI)) either 10 min after the BL session (DELAY, n = 4) or 10 min before the EXT session (IMMED, n = 4) on Day 2 in another context (context B). On Day 2, all rats received 50 tone-alone presentations during the extinction session (EXT, context A). On Day 3, all rats were returned to the extinction context (context A) again and exposed to another 10 CS-alone presentations for the retention test (TEST). Freezing was assessed during the BL, COND, EXT, and TEST session.

### Histology

At the end of experiments, anodal current (20 µA, 20 s) was passed through the tetrode tips to create small marking lesions. Rats were then perfused across the heart with 0.9% saline followed by a 10% formalin solution. After extraction from the skull, brains were post-fixed in 10% formalin solution for two days, at which time the solution was replaced with a 10% formalin and 30% sucrose solution until sectioning. Sections (45 µm thick) were cut on a cryostat (−20°C), and wet-mounted on glass microscope slides with 70% ethanol. After drying, the sections were stained with 0.25% thionin for visualization of lesions.

### Data analysis

All behavioral data are expressed as means and standard error of the means (SEM) and were submitted to analysis of variance (ANOVA) in 10-trial blocks during BL, early/late EXT (first and last 10 trials of the EXT session), and TEST sessions unless specified otherwise. *Post hoc* comparisons in the form of Fisher's PLSD tests were performed after a significant F ration.

Tone-evoked responses for each single unit were summed across 10 CS trials in different behavioral phases and post-CS activity was normalized to the 2s pre-CS baseline (200ms bin-size) to a generate standard score (*z*-score) during the 2-sec CS period. A burst was defined as three or more consecutive spikes with an interval of less than 30 ms between the first two spikes and less than 50 ms in subsequent spikes.
